# Effective Removal of Fe (III) from Strongly Acidic Wastewater by Pyridine-Modified Chitosan: Synthesis, Efficiency, and Mechanism

**DOI:** 10.3390/molecules28083445

**Published:** 2023-04-13

**Authors:** Lei Zhang, Heng Liu, Jiaqi Zhu, Xueling Liu, Likun Li, Yanjun Huang, Benquan Fu, Guozhi Fan, Yi Wang

**Affiliations:** 1 School of Chemistry and Environmental Engineering, Wuhan Polytechnic University, Wuhan 430023, Chinawangyi2020@whpu.edu.cn (Y.W.); 2 China-Ukraine Institute of Welding, Guangdong Academy of Sciences, Guangzhou 510650, China; 3 R & D Center of Wuhan Iron and Steel Company, Wuhan 430080, China

**Keywords:** acidic wastewater, heavy metal ions, pyridine-modified chitosan adsorbent, adsorption mechanism

## Abstract

A novel pyridine-modified chitosan (PYCS) adsorbent was prepared in a multistep procedure including the successive grafting of 2-(chloromethyl) pyridine hydrochloride and crosslinking with glutaraldehyde. Then, the as-prepared materials were used as adsorbents for the removal of metal ions from acidic wastewater. Batch adsorption experiments were carried out to study the impact of various factors such as solution pH value, contact time, temperature, and Fe (III) concentration. The results showed that the absorbent exhibited a high capacity of Fe (III) and the maximum adsorption capacity was up to 66.20 mg/g under optimal experimental conditions (the adsorption time = 12 h, pH = 2.5, and T = 303 K). Adsorption kinetics and isotherm data were accurately described by the pseudo-second-order kinetic model and Sips model, respectively. Thermodynamic studies confirmed that the adsorption was a spontaneous endothermic process. Moreover, the adsorption mechanism was investigated using Fourier transform infrared spectroscopy (FTIR) and X-ray photoelectron spectroscopy (XPS). The results revealed the pyridine group forms a stable chelate with iron (III) ions. Therefore, this acid-resistant adsorbent exhibited excellent adsorption performance for heavy metal ions from acidic wastewater compared to the conventional adsorbents, helping realize direct decontamination and secondary utilization.

## 1. Introduction

A large amount of strongly acidic wastewater is produced during the flue gas desulfurization process in the coking industry. This type of wastewater has a high concentration of sulfuric acid (1–3wt%) and contains high concentrations of iron ions and traces of arsenic, lead, and mercury ions [1,2]. It is reported that about 5.0–7.4 million tons of strongly acidic wastewater are generated from the coking industry per year in China [3]. The most common practice is to add an alkali for neutralization and formation of hydroxide precipitants, which can be separated by coagulation/flocculation, precipitation, adsorption, and filtration methods [4]. However, this process needs further improvements due to generating large amounts of solid hazardous waste, which poses serious environmental risks. More recently, the recycling of acidic wastewater as dilute sulfuric acid for chemical production of, for example, (NH_4_)_2_SO_4_ is considered a reliable method [5,6]. Because the high concentration of heavy metals will affect the quality of downstream products, heavy metal ions in acidic wastewater should be effectively removed.

In recent years, various technologies have been employed to remove heavy metal ions from acidic wastewater, such as membrane filtration, adsorption, solvent extraction, and ion exchange [7,8,9,10]. Compared with other techniques, adsorption is considered an effective and promising method in terms of low cost, easy operation, and high efficiency [11]. Recently, the development and utilization of inexpensive and eco-friendly biopolymers have attracted great attention. Chitosan is a linear polysaccharide extracted from chitin, which is the second most abundant natural polymer; it can be found in the crustacean shells of shrimps, crabs, and lobsters [12,13]. The hydroxyl and amino functional groups on the backbone of chitosan can serve as coordination sites to form complexes with various heavy metal ions [14]. Nonetheless, water solubility under acidic conditions and low adsorption capacity restricts chitosan as an efficient adsorbent [15]. Therefore, the modification of chitosan is of great significance to enhance its adsorption performance.

According to the reports, chitosan is modified by various functional groups for heavy metals removal, such as ethylenediamine, polyaniline, α-ketoglutaric acid, ionic liquid [16,17,18,19], etc. However, the removal efficiency of most chitosan-based adsorbents decreases significantly at lower pH, which limits their application in strong acidic wastewater. Pyridine is an excellent basic ligand with nitrogen donor atoms. Due to the electron absorption effect of aromatic group, the nitrogen atom of pyridine group exhibits a low pKa, which makes it difficult to protonate. Therefore, pyridine groups can combine with heavy metal ions and form complexes at very low pH value [20,21]. The introduction of pyridine groups into chitosan structure will enhance its adsorption performance in strongly acidic solutions.

In this work, a pyridine-modified and glutaraldehyde-crosslinked chitosan (PYCS) was prepared and employed for the removal of Fe (III) from strongly acidic wastewater. The obtained adsorbent PYCS was characterized using Fourier transform infrared spectroscopy (FTIR), scanning electron microscopy (SEM), thermogravimetric analysis (TGA), and an X-ray diffractometer (XRD). The effects of parameters such as pH value, contact time, initial Fe (III) concentration, and temperature on adsorption behaviors were investigated. Isotherm, kinetic, thermodynamics, and inter-particle diffusion models were used to evaluate the adsorption process. Furthermore, the reusability of PYCS was studied in an adsorption–desorption experiment. Finally, the adsorption mechanism of Fe (III) on PYCS was clarified using FTIR and XPS.

## 2. Results and Discussion

### 2.1. Characterization

**Figure 1:**
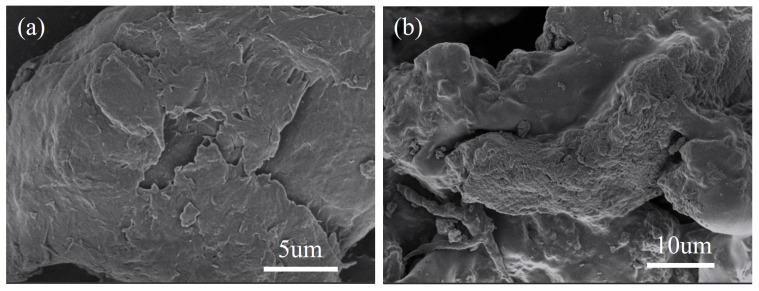
SEM images ((**a**): CS, (**b**): PYCS).

Figure 1 shows the SEM images of chitosan (CS) and pyridine-modified chitosan (PYCS). The pristine CS exhibited a smooth surface and layered structure (Figure 1a) while the CS modified with pyridine displayed some irregular aggregates with a loose honeycomb structure (Figure 1b). The flake-like particles were formed by glutaraldehyde crosslinking chitosan. The surface with plenty of irregular pores increased the surface area, indicating abundant binding sites for metal ion adsorption.

**Figure 2:**
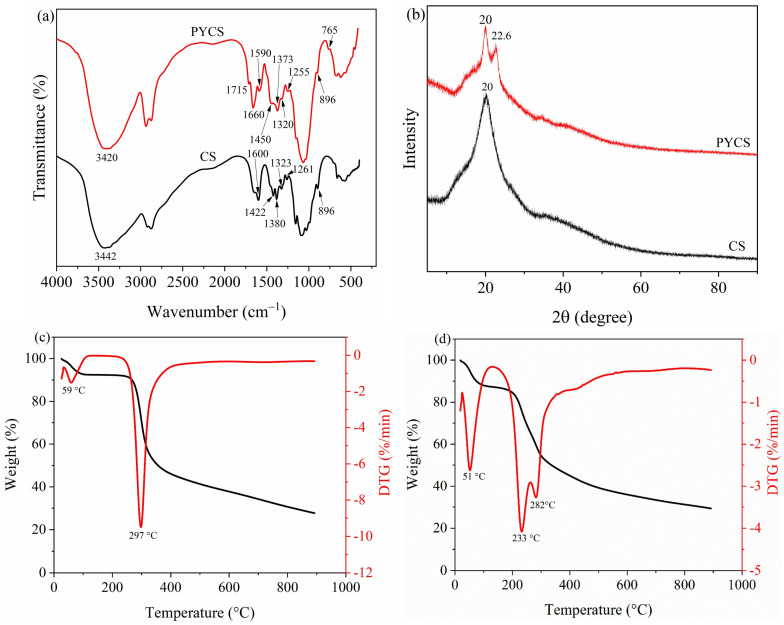
(**a**) FTIR spectra, (**b**) XRD spectra, and TGA–DTG curves ((**c**): CS, (**d**): PYCS).

In order to detect the chemical bonds of modified chitosan, FTIR spectroscopy of CS and PYCS materials were detected at 4000 cm^−1^~400 cm^−1^, and the results are shown in Figure 2a. The strong and broad peaks at 3420 cm^−1^ and 3442 cm^−1^ represent the absorption peaks of −OH and −NH_2_, which overlap to produce multiple broadened absorption peaks [22]. The CS had a strong absorption peak at 1600 cm^−1^, which was caused by the N−H in-plane bending vibration of the primary amine, and the vibration peak disappeared after modification [23]. After modification with pyridine, a new absorption peak near 1660 cm^−1^ was the characteristic C=N peak on the pyridine ring and C=C. In addition, the new absorption peak of skeletal vibration of pyridine occurred at 1590 cm^−1^ and 1450 cm^−1^, and the adsorption feature near 765 cm^−1^ was caused by the =C−H bending vibration of pyridine ring. The above analysis showed that pyridine group was successfully grafted onto CS. The characteristic absorption peak of β-D pyranoside near 900 cm^−1^ still existed, indicating that the grafting reaction did not destroy the pyran ring of chitosan.

Figure 2b presents the XRD spectra of CS and PYCS. The CS showed a broad peak at 20.0°, indicating the presence of amorphous regions and regular crystalline regions [24]. When pyridine was grafted into chitosan via the substitution reaction, the intensity of the diffraction peak at 20.0° decreased, indicating the decrease in crystallinity. The crystallinity of PYCS decreased due to the destruction of the sequence of chitosan molecular chains by the crosslinking reaction. According to Thien et al. [25], the typical functional groups of modified CS would become more active and flexible in the amorphous state, which would enable PYCS to effectively form a complex with many metal ions.

The TGA–DTG curves of CS and PYCS are shown in Figure 2c,d. It can be seen from the figure that the TG curves of CS and PYCS are similar, indicating that they have similar skeleton structures. The thermal profile of CS was divided into two stages: the first peak of CS at 59 °C, where the weight loss rate was approximately 7.50%, which was due to water loss [26]. The second stage was the rapid weight loss degradation stage, which occurred at 215−550 °C, and the peak weight loss of CS in the second stage was at 297  °C. The weight loss rate was 52.04%, which was caused by the breakage of glycosidic bonds and the degradation of the structure of chitosan itself [27]. However, the thermal weight loss of PYCS occurred at three unique peaks, specifically, 51 °C, 233 °C, and 282 °C with varied percentage weight reductions of 11.96%, 27.47%, and 28.38%, respectively. The first stage of mass loss was due to the release of typically strong hydrogen-bonded water [28]. The thermal degradation at 130−260 °C observed in the PYCS may have been due to the elimination of the crosslinker by the breakage of the ionic interaction between the CS and the glutaraldehyde. Regarding the thermal degradation observed in cases at 260−550 °C, this stage was the result of glycosidic bond cleavage, main chain degradation, and concomitant graft decomposition [29]. These results indicated that the thermal stability of PYCS was lower than that of CS, which may be caused by the destruction of intramolecular and intermolecular hydrogen bonds of chitosan by the newly introduced side chains [30].

### 2.2. Effect of pH on Adsorption

**Figure 3:**
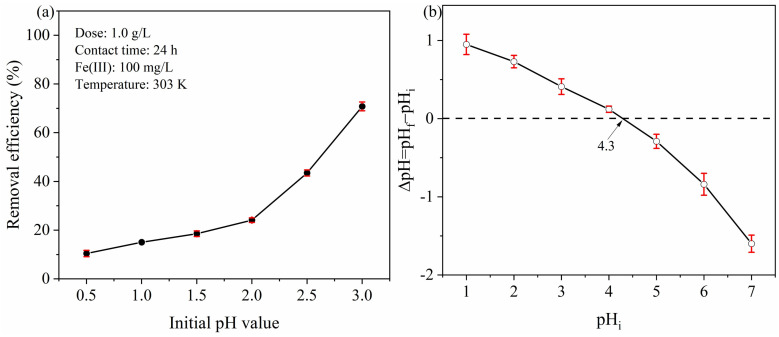
Effect of solution pH value on Fe (III) (**a**) removal efficiency and (**b**) the point of zero charge of PYCS.

The pH value of solution is one of the most important factors for adsorption of PYCS, because it influences the ionization state of functional groups [31,32]. The pH should be set below 3 because of the metal precipitation at higher pH levels is not in line with the real wastewater quality. The Fe (III) adsorption experiments were performed at different pH values (from 0.5 to 3). It can be seen from Figure 3a that with the increase in pH value, the removal efficiency of Fe (III) increased significantly. The overall trend was increasing, but it was not obvious when the acid was strong. The reason was that the N atom on the pyridine ring was protonated and positively charged in extremely acidic solution [33], which weakened its chelating ability with Fe (III). The lower the pH, the more evident the protonation, thus leading the removal efficiency to rise slowly [34]. When the pH was 3, the removal efficiency was significantly improved, mainly due to part of Fe (III) precipitation. Additionally, the pH_PZC_ value of PYCS adsorbent was examined to be 4.3 using the pH drift method (Figure 3b). When the solution pH value was less than pH_PZC_, the surface of the PYCS adsorbent was positively charged, which was not conducive to Fe (III) adsorption. Based on the precipitation of Fe (III) at a pH greater than 2.7, the solution pH was chosen to be 2.5 in the subsequent experiments.

### 2.3. Adsorption Kinetics

**Figure 4:**
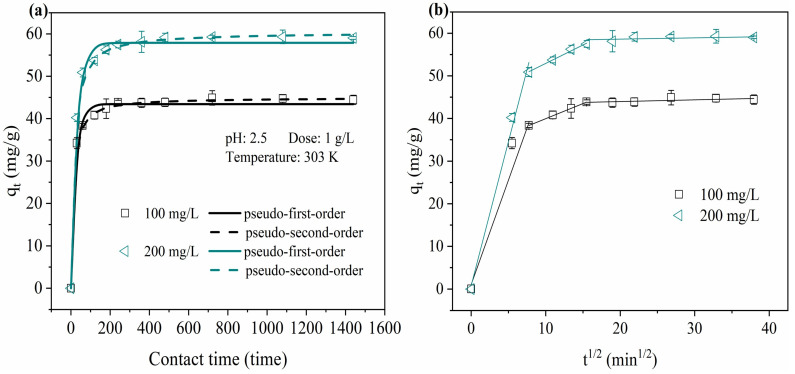
Kinetic profiles of Fe (III) adsorption by PYCS: (**a**) The pseudo-first- and pseudo-second-order kinetic plots. (**b**) The intra-particle diffusion kinetic model.

As plotted in Figure 4a, the uptake capacity of Fe (III) increased as the contact time continued, and gradually reached adsorption equilibrium. PYCS had many free active sites, and the driving force for adsorption was very strong initially. The equilibrium concentration of Fe (III) decreased with time as the number of free active sites adsorbed declined, then the adsorption rate gradually decreased, and finally tended to saturate. The data first demonstrated that the time varied greatly for the adsorption of different Fe (III) concentrations to reach equilibrium, with values of 400 and 720 min for 100 and 200 mg/L, respectively. In order to reach the adsorption equilibrium sufficiently, the contact time of 12 h was selected in the following isotherm experiments.

To investigate the rate-limiting step in the adsorption mechanisms, the kinetic experimental data were fitted with Lagergren’s pseudo-first-order, Ho’s pseudo-second-order, and intra-particle diffusion kinetic models [35,36,37]. The nonlinear form equations for the three models were expressed as follows in Equations (1)–(3).

The pseudo-first-order kinetic model is expressed by

(1) $$q_{t}=q_{e}[1-e^{-k_{1}t}]$$

The pseudo-second-order kinetic model is expressed by

(2) $$q_{t}=\frac{q_{e}^{2}k_{2}t}{1+k_{2}q_{e}t}$$

The intra-particle diffusion kinetic model is expressed by

(3) $$q_{t}=k_{i}t^{1/2}+C$$

where *q_t_* and *q_e_* were the adsorption capacities (mg/g) of adsorbents at contact time *t* (min) and equilibrium, respectively. Whereas, *k*_1_ (min^−1^) and *k*_2_ (g/mg·min) were the rate constants of the pseudo-first-order and pseudo-second-order kinetic models, respectively. *k_i_* (mg/g·min^1/2^) was the rate constant for the intra-particle diffusion model, and *C* was the thickness of boundary layer [38,39].

In this work, the root mean square error (RMSE) was used to measure the value deviation between the experimental data and the predicted values. In principle, the lower the RMSE value, the more suitable the model fits. RMSE could be calculated using the following equation:

(4) $$RMSE=\sqrt{\frac{1}{n}\sum_{i=1}^{n}\left(q_{cali}-q_{\exp i}\right)}$$

where *q_cali_* and *q*_exp*i*_ were the predicted and measured values of the adsorption capacity at time *t*, respectively, and *n* was the number of experimental data.

The experimental data for the adsorption kinetics were fitted using the non-linear form of the pseudo-first-order and pseudo-second-order models, as shown in Figure 4a, and the values of kinetic parameters and their RMSE values are summarized in Table 1. Much better fitting results were obtained when the pseudo-second-order model was adopted for the adsorption kinetics of the different Fe (III) concentrations by PYCS. In addition, the theoretical adsorption capacity predicted by the pseudo-second-order model was much closer to the experimental value. These results showed that the pseudo-second-order model was more suitable for describing the adsorption process than the pseudo-first-order model, which indicated that the rate-determining step of PYCS may be a chemisorption process.

The intra-particle diffusion model is further applied to determine the rate-controlling step of the adsorption processes. According to Figure 4b, it can be observed that the three adsorption curves were divided into three linear parts and did not pass through the origin. The modeling results indicated that the adsorption process was mainly controlled by intra-particle diffusion, as well as other mechanisms including surface chemisorption process. The first stage of the straight line represented the boundary layer diffusion process of Fe (III) adsorption by PYCS. The next stage represented the diffusion of Fe (III) into PYCS channels and the process of gradual adsorption (intra-particle diffusion). The slope of the third stage was almost zero, indicating the equilibrium stage of adsorption and desorption [40,41,42]. Simultaneously, the diffusion rate constants followed the order of *k*_i,1_ > *k*_i,2_ > *k*_i,3_ which may be affected by the change in the number of PYCS diffusion sites. The rate constant in the first stage was much higher than that in other stages, which meant that the film diffusion was the main rate-limiting step in the whole process, but the adsorption rate was also affected and controlled by the external diffusion step and the adsorbent surface diffusion.

### 2.4. Adsorption Isotherm

**Figure 5:**
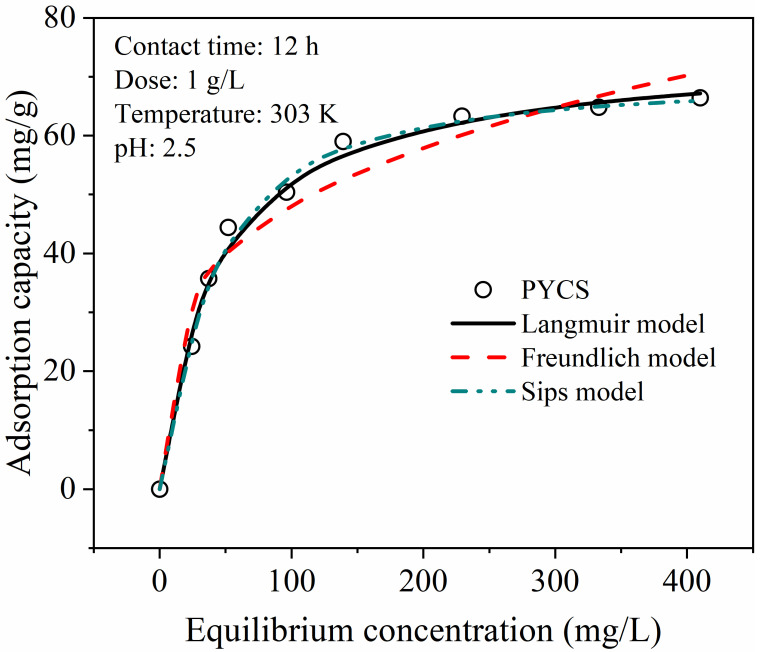
Adsorption isotherms of Fe (III) on PYCS.

Adsorption isotherm is usually used to study the interaction between the adsorbent and pollutant in equilibrium, and can also estimate the maximum adsorption capacity of the adsorbent for further comparison with adsorbents reported in other studies. As can be seen from Figure 5, with the increase in the initial Fe (III) concentration, the adsorption of Fe (III) by PYCS gradually increased and reached surface saturation at high concentration. This was because at a lower initial Fe (III) concentration, PYCS adsorption active sites were enough to hold the adsorbate, and the interaction sites were not saturated. As the concentration of Fe (III) increased, almost all active sites on the surface of the adsorbent were occupied, leaving the Fe (III) uptake unchanged, then the adsorption capacity for Fe (III) would reach the maximum. In this study, the maximum adsorption capacity of PYCS was 66.20 mg/g.

To investigate the adsorption behavior and mechanism of PYCS, the three common adsorption isotherm models were employed to fit the experimental data, and their nonlinear equations were given as follows.

The Langmuir isotherm model assumes that monolayer adsorption occurs on the adsorbent surface with equivalent adsorption sites, and there is no interaction between the adsorbed pollutants [43,44].

(5) $$q_{e}=\frac{q_{m}K_{L}C_{e}}{1+K_{L}C_{e}}$$

The Freundlich equation is one of the earliest empirical equations used to describe equilibrium data and adsorption characteristics for a heterogeneous surface [45].

(6) $$q_{e}=K_{F}C_{e}^{1/n_{F}}$$

The Sips model is a hybrid of the Langmuir and the Freundlich isotherms [46].

(7) $$q_{e}=\frac{q_{m}{\left(K_{S}C_{e}\right)}^{n_{S}}}{1+{\left(K_{S}C_{e}\right)}^{n_{S}}}$$

where *q_e_* is the amount of Fe (III) adsorbed at equilibrium (mg/g), and *q_m_* is the maximum adsorption capacity of Fe (III) on PYCS (mg/g); *C_e_* represents the equilibrium concentration of Fe (III) (mg/L); *K_L_* is the Langmuir isothermal constant which is related to the affinity of binding sites (L/mg). *K_F_* is the Freundlich isothermal constant (mg^(1−n)^·L^n^/g), and *n_F_* is the heterogeneity factors. *K_S_* is the Langmuir equilibrium constant (L/mg), and *n_S_* is comparable to the Freundlich heterogeneity factor (*n_S_* = 1/*n_F_*).

The adsorption isotherm parameters simulated from the three isotherm models are summarized in Table 2. Based on the value of the RMSE, the adsorption isotherms of Fe (III) were fitted better by the Sips model. In addition, the heterogeneity factor n_S_ values shown in Table 2 were more than unit, and the experimental (*q*_m, exp_) value was in good agreement with calculated (*q*_m, cal_) value, indicating that the adsorption of Fe (III) ions onto the prepared adsorbent was a heterogeneous process [40,41,42]. Table 3 lists the maximum adsorption capacities (*q*_m_) of Fe (III) on PYCS with various adsorbents. It can be seen that the maximum adsorption capacity of PYCS was higher than the other adsorbents listed in Table 4. Nevertheless, the maximum adsorption capacity of PYCS was measured at pH 2.5, while the maximum adsorption capacities of other adsorbents were measured at pH greater than 3. This showed that PYCS displayed high adsorption capacity, which is beneficial to remove Fe (III) from strong acid solution.

### 2.5. Adsorption Thermodynamics

The temperature is an essential factor affecting the adsorption effect. Gibbs free energy (Δ*G*), enthalpy (Δ*H*), and entropy (Δ*S*) are important parameters reflecting the thermodynamic reaction. The thermodynamic relations are depicted in the following equations [54,55].

(8) $$K_{d}=q_{e}/C_{e}$$

(9) ΔG=ΔH−TΔS

(10) $$\ln K_{d}=\Delta S/R-\Delta H/RT$$

where *R* (8.314 J/(mol·K)) is the ideal gas constant; *K*_d_ is the adsorption equilibrium constant; *T* (K) is the experimental temperature; Δ*S* (J/(mol·K)), Δ*H* (kJ/mol), and Δ*G* (kJ/mol) refer to entropy change, enthalpy change, and Gibbs free energy change, respectively.

The calculation results of thermodynamic equilibrium coefficient and thermodynamic parameters of PYCS adsorption process are shown in Table 4. The Δ*H* values were positive, which indicated that the adsorption process was endothermic. Meanwhile, the Δ*G* values were negative, thus indicating that the adsorption process was spontaneous. Δ*S* values were positive which reveals that the reaction system was chaotic and that Fe (III) had good contact with PYCS. Therefore, the reaction between PYCS and Fe (III) was a spontaneous endothermic process, and increasing the temperature can make this reaction proceed more efficiently.

### 2.6. Regeneration and Reusability

**Figure 6:**
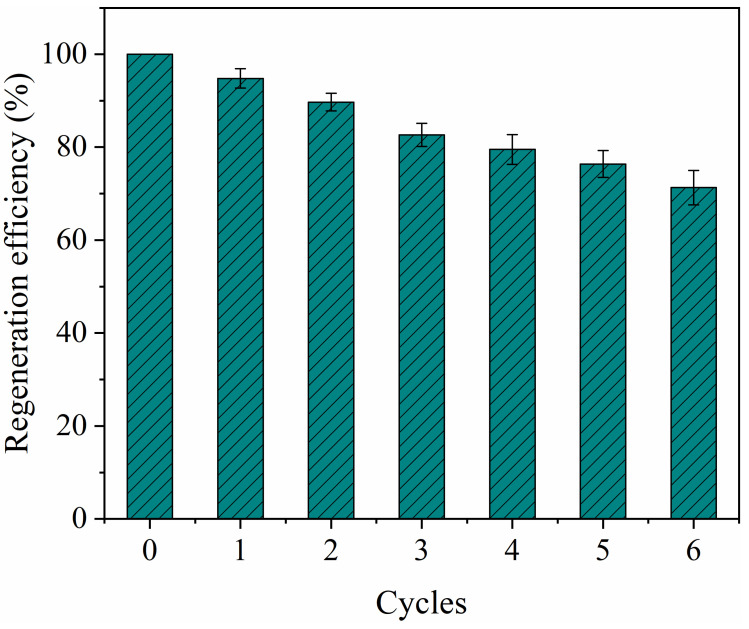
Reuse of PYCS in removal of Fe (III) from acidic wastewater.

As the reusability of the adsorbent was very important for its industrial application, the adsorption–desorption cycles were conducted six times to further evaluate the reusability of the as-prepared PYCS. As observed from Figure 6, the adsorption ability slightly decreased as the number of cycles increased. After six cycles, the adsorption capacity of PYCS was maintained at 71% of that of the first cycle. The decrease of adsorption capacity may be the loss or degradation of adsorbent [39]. In addition, the incomplete removal of Fe (III) ions in the adsorbent by the eluent may be another reason for the reduction of adsorption capacity. Overall, PYCS showed a good reusability and had great potential as an efficient adsorbent for the removal of Fe (III) ions from polluted water.

### 2.7. Application in Real Acidic Wastewater

**Figure 7:**
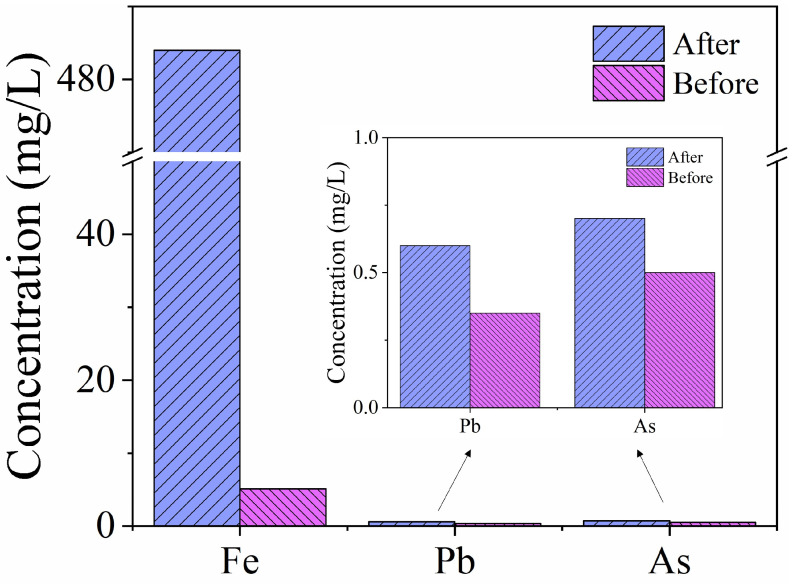
The removal of metal ions from real wastewater.

The prepared materials were used for the adsorption of heavy metals in real acidic wastewater to evaluate the practical applicability. The results are in Figure 7. It can be found that the removal rates of Fe (III), Pb (II), and As (III) were 98.9%, 34.5%, and 28.6%, respectively. The results proved PYCS as a potential material for heavy metal removal in water treatment with high efficiency.

### 2.8. Adsorption Mechanism

**Figure 8:**
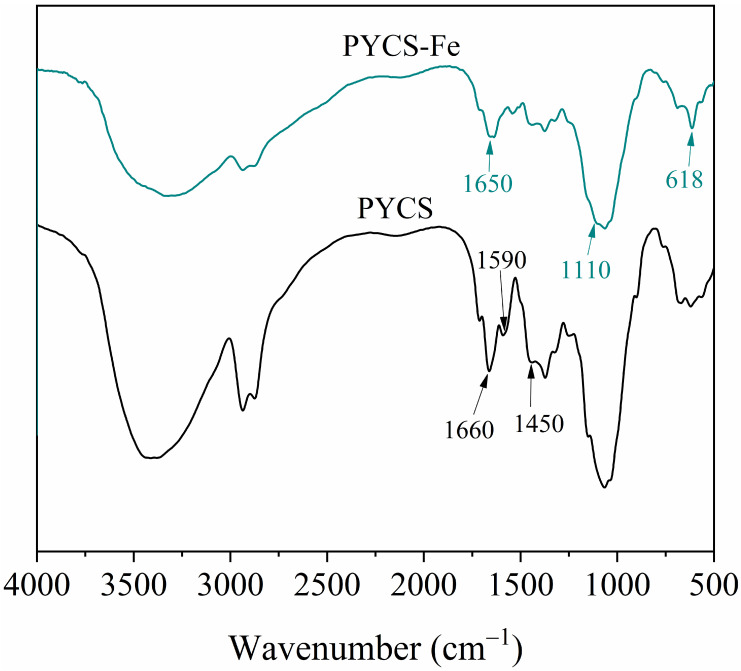
FTIR spectra of PYCS before and after adsorbing Fe (III).

The adsorption mechanism of PYCS was investigated by examining the XPS and FT-IR data. The FT-IR spectra of the PYCS before and after loading with Fe (III) are shown in Figure 8. For PYCS, the adsorption band near 1450 cm^−1^ corresponded to the stretching vibrations of C−N, and the adsorption feature near 1590 cm^−1^ was caused by skeletal vibration of pyridine. After adsorption, the strength of the C=N (1660 cm^−1^) bonds reduced and moved at a minor wave length, confirming that pyridines were the functional groups of PYCS, forming coordinate bonds with Fe (III) [56]. The stretching vibration of pyridine at 1590 cm^−1^ and that of C−N at 1450 cm^−1^ were significantly weakened and almost disappeared after adsorption, indicating that pyridine was involved in the chelating interaction with Fe (III). The appearance of new peaks at 1110 cm^−1^ and 618 cm^−1^ corresponded to the stretching vibration of $SO_{4}^{2-}$ ions after adsorption [20].

**Figure 9:**
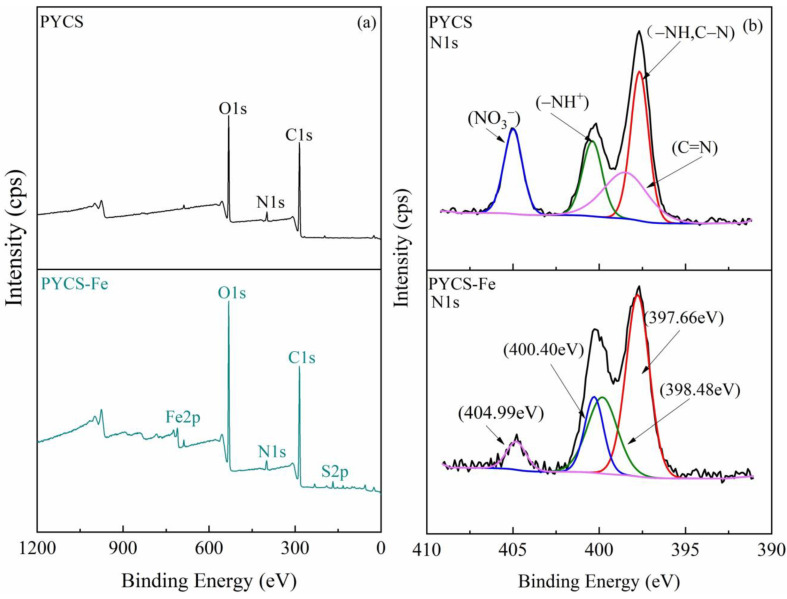
Characterization of PYCS before and after Fe (III) adsorption: (**a**) XPS wide scan spectra. (**b**) XPS spectra of N1s.

To further distinguish the species of functional groups and confirm the coordination interaction between nitrogen atoms and Fe (III), XPS characterization of PYCS before and after Fe trapping were performed. The XPS wide scans and N1s spectra of PYCS are exhibited in Figure 9. For the raw PYCS, the N1s core-level XPS spectra were deconvoluted into 4 peaks at 397.66, 400.40, 398.48, and 404.99 eV, which corresponded to the nitrogen atoms in the neutral amine (−NH or C−N), protonated amine (−NH^+^), pyridine (C=N), and nitrate ions (${\mathrm{NO}}_{3}^{-}$), respectively [57,58,59,60]. Meanwhile, the existence of nitrate ions was due to the synthetic PYCS cleaned by HNO_3_, which was consistent with the results of FT-IR. After the adsorption of Fe (III) on PYCS at pH 2.5, the minor shift for −NH or C−N, and the obvious shift for C=N for PYCS before and after the adsorption of Fe (III) may be interpreted such that the pyridine nitrogen (C=N) atoms in the organic functional groups on the surface of PYCS had coordinative chelation with Fe (III) during the adsorption process [61]. In addition, the peak value of nitrate ions decreased significantly at 404.99 eV, which can prove that nitrate ions were replaced by sulfate ions.

**Figure 10:**
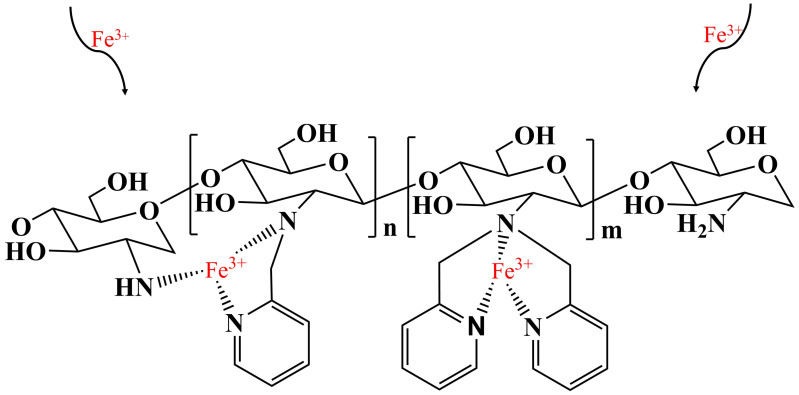
Possible mechanism of the adsorption process.

The possible mechanism is shown in Figure 10. The adsorbent PYCS was capable of capturing Fe (III) through nitrogen atoms of pyridine that participated in the chelating effect at pH 2.5. According to literature reports, the polyamine adsorbent lost its adsorption ability below pH 3 [62], but pyridine-modified adsorbent could adsorb Fe (III). Owing to the weak alkalinity of the aliphatic amines and pyridine ring, competitive adsorption would occur between Fe (III) ions and H^+^ under low pH. Hence, the active adsorption sites of the nitrogen atoms would be partially protonated, resulting in lower adsorption capacity.

## 3. Materials and Methods

### 3.1. Chemical and Materials

Chitosan (>95% deacetylated, 100–200 mPa s, M.wt%. 6.9 × 10^5^) was supplied from the Aladdin Industrial Corporation (Shanghai, China). Acetic acid (CH_3_COOH, AR), sodium hydroxide (NaOH, AR), and 2-Chloromethylpyridine hydrochloride (C_6_H_6_ClN·HCl, AR) were all purchased from Macklin Biochemical Co., Ltd. (Shanghai, China). Ethanol (CH_3_CH_2_OH, AR), glutaraldehyde, sodium carbonate (Na_2_CO_3_, AR), nitric acid (HNO_3_, AR), ferric sulfate (Fe_2_(SO_4_)_3_, AR), sulfuric acid (H_2_SO_4_, AR), and hydrochloric acid (HCl, AR) were supplied by Sinopharm Chemical Reagent Co., Ltd. (Shanghai, China), all reagents of which were analytical grade and used directly without further purification. All water mentioned in this study was deionized water (18.2 MΩ cm). The metal concentrations in as-pretreated real acidic wastewater from an iron and steel industry in Central China, were determined, as listed in Table 5.

### 3.2. Preparation of Pyridine-Modified Chitosan

The preparation procedures of pyridine-modified chitosan (PYCS) adsorbent were as follows: Firstly, 1 g of chitosan was dissolved in 50 mL of acetic acid (2% *v*/*v*). Then, 250 mL of NaOH (0.5 mol/L) was slowly added dropwise to form chitosan beads. The mixture was stirred for 3 h. After separation, the particles were washed to neutrality with deionized water and ethanol.

Then, the as-obtained chitosan beads, 3.06 g 2-(chloromethyl) pyridine hydrochloride (dissolved in 50 mL ethanol), and 0.83 g sodium carbonate were introduced into a 250 mL round-bottom flask. The reaction was carried out through reflux condensing at 363 K for 24 h. After filtration, the product was immersed into a mixture solution containing 2.5wt% glutaraldehyde for 12 h at 303 K with continuously shaking, then rinsed with ethanol and deionized water.

**Figure 11:**
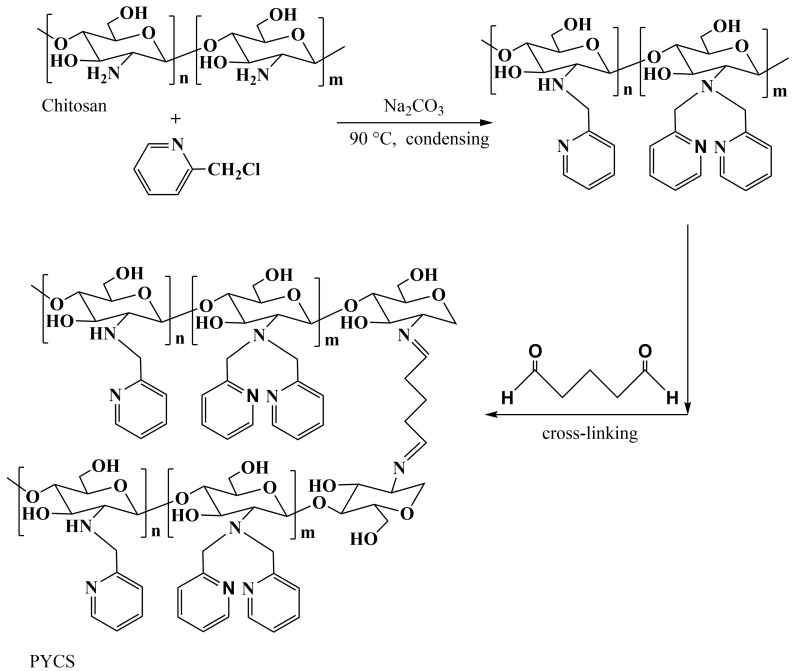
Synthetic procedure of PYCS.

After drying in vacuum at 313 K for 12 h, the adsorbent PYCS was obtained. The synthetic procedure of PYCS is shown in Figure 11.

### 3.3. Characterization

The chemical constituents of samples were recorded using the Fourier transform infrared spectrometer (FTIR, Nicolet iS10, ThermoFisher Scientific, Waltham, MA, USA) in a range of 4000 to 400 cm^−1^. The surface morphology of the samples was characterized using a scanning electron microscope (SEM, S4800, Hitachi, Tokyo, Japan). Thermal decomposition characteristic analysis was carried out on a thermogravimetric analyzer (TGA, STA 7300, Hitachi, Tokyo, Japan) at a heating rate 10 °C/min from room temperature to 900 °C in a N_2_ flow. The X-ray photoelectron spectroscopy (XPS, K-Alpha, Thermo Scientific, Waltham, MA, USA) was used to investigated the surface element component of the samples. The crystallinity of the samples was characterized on the X-ray diffractometer (XRD, XRD-7000, Shimadzu, Kyoto, Japan) over the 2 thetas range of 5°–90°. The concentration of each heavy metal ion was determined using inductively coupled plasma mass spectrometry (ICP, iCAP Q, Thermo Fisher Scientific, Waltham, MA, USA). The pH of the zero charge (pH_pzc_) of the prepared PYCS was determined using the batch equilibrium method [63].

### 3.4. Adsorption Experiments

Batch adsorption tests were undertaken using a centrifuge tube at 303 K. In general, 0.02 g of PYCS and 20 mL aqueous solution containing Fe (III) were added to the centrifuge tube and agitated at 170 rpm on a shaker. To examine the effect of pH on Fe (III) removal, the adsorption was conducted at the Fe (III) concentration of 100 mg/L in the pH range of 0.5–3.0 for 24 h. The initial pH of Fe (III) aqueous solution was measured by pH meter and was adjusted by using 0.1 mol/L H_2_SO_4_ and NaOH (aq).

For adsorption kinetic experiments, 0.02 g of PYCS was added into 20 mL of an aqueous solution containing Fe (III). The initial pH and Fe (III) concentration value of the solution were 2.5, 100 mg/L, respectively. The adsorption isotherm was investigated at 303 K, and the initial concentration of the Fe (III) varied from 20 to 500 mg/L at pH 2.5. To evaluate adsorption thermodynamics, 303 K, 313 K, 323 K, and 333 K were selected for the adsorption. After adsorption, the reaction solution was sampled and immediately filtered through a 0.45 μm membrane filter. Finally, the residual Fe (III) concentration in the wastewater was determined using ICP-MS. The adsorption capacity of adsorbent was calculated using the following Equation.

(11) $$q_{e}=\frac{(C_{0}-C_{e})V}{m}$$

where *q_e_* (mg/g) represents the amount of Fe (III) adsorbed onto the adsorbent at equilibrium, *C*_0_ and *C_e_* (mg/L) are the initial and equilibrium concentration of Fe (III) ions, respectively, *V* (L) represents the volume of solution, and *m* (g) represents dosage of adsorbent.

### 3.5. Regeneration Studies

In order to evaluate the regeneration and reuse of PYCS, 0.02 g PYCS was first added to 20 mL of a solution containing 100 mg/L of Fe (III) ions, wherein the solution pH was 2.5, and then was agitated at 170 rpm for 24 h at 303 K. After the adsorption process, the absorbent saturated with Fe (III) was filtered and washed with water to remove the un-adsorbed Fe (III), then agitated with 20 mL of elution solution (3 mol/L HCl) for another 24 h. The regenerated PYCS was filtered and washed with water several times and then was stored for the next experiment. The adsorption regeneration experiment was repeated 6 times.

### 3.6. Application in Real Acidic Wastewater

In this work, PYCS was used to treat real acidic wastewater from a coking plant in Wuhan. The detailed metal concentration of the real acidic wastewater is listed in Table 5. 1 g PYCS was added into 20 mL of real acidic wastewater and agitated at 170 rpm for 24 h at 303 K. Afterwards, the solution samples were filtrated with 0.45 μm filters, and the residual Fe (III) concentration in the solution was analyzed using ICP-MS.

## 4. Conclusions

A pyridine-modified chitosan (PYCS) was successfully prepared by modifying pyridine onto chitosan, which was then used to remove Fe (III) from acidic wastewater. The adsorbent has a distinguished adsorption effect for Fe (III), and the removal rates of Fe (III), Pb (II), and As (III) in real acidic wastewater were 98.9%, 34.5%, and 28.6%, respectively. The maximum adsorption capacity was 66.20 mg/g, which was better than most reported adsorbents. Comprehensive adsorption kinetics and isotherm results showed that adsorption was heterogeneous chemisorption process. Fe (III) adsorption on PYCS was endothermic and spontaneous in nature. In addition, after 6 cycles of adsorption, the adsorption capacity of the material could still reach 71%, which indicated that it had important practical application value. In short, the material may be used as a promising, efficient, and environmentally friendly adsorbent for the remediation of heavy metal pollution.

## Figures and Tables

**Table 1:** Kinetic parameters and RMSE values for adsorption of Fe (III) on PYCS.

Kinetic Models	Parameters	Concentration (mg/L)	
		100	200
Pseudo-first-order	*q*_e,exp_ (mg/g)	44.88	59.29
	*q*_e,cal_ (mg/g)	43.43	57.87
	*k*_1_ (min^−1^)	0.047	0.037
	RMSE	1.36	1.47
Pseudo-second-order	*q*_e,cal_ (mg/g)	44.96	60.38
	*k*_2_ (g/mg·min)	0.0022	0.0012
	RMSE	0.38	0.72
Intra-particle diffusion	*k*_i,1_ (mg/g·min^1/2^)	4.90	6.75
	*k*_i,2_ (mg/g·min^1/2^)	0.71	0.86
	*k*_i,3_ (mg/g·min^1/2^)	0.04	0.03
	*C*_i,1_ (mg/g)	1.10	0.86
	*C*_i,2_ (mg/g)	32.89	44.30
	*C*_i,3_ (mg/g)	43.19	58.03

**Table 2:** Isothermal model constants, RMSE values and adsorption capacities of PYCS.

Isothermal Models	Parameters	
Langmuir	*q*_m,exp_ (mg/g)	66.20
	*q*_m,cal_ (mg/g)	73.83
	*K*_L_ (L/mg)	0.024
	RMSE	1.76
Freundlich	*n*_F_	3.70
	*K*_F_ (mg^(1−n)^ L^n^/g)	13.93
	RMSE	4.33
Sips	*q*_e,cal_ (mg/g)	68.93
	*n*_S_	1.27
	*K*_S_ (L/mg)	0.027
	RMSE	1.41

**Table 3:** Comparison of maximum adsorption capacities of Fe (III) on PYCS with other adsorbents.

Adsorbent	Conditions		Adsorption Capacity (mg/g)	Reference
	pH	T (K)		
Chitosan/attapulgite	3	308	47.17	[47]
Chitosan/MMT	5.5	298	7.03	[48]
Chitosan films	4.5	298	299.04	[49]
Chitosan/PVA	3	298	136	[50]
Carboxymethylated chitosan	4.7	298	18.5	[51]
Chitosan-EGDE	3	298	46.30	[52]
Amine-modified chitosan resins	2.5	298	109.61	[53]
PYCS	2.5	303	66.20	This work

**Table 4:** Thermodynamic parameters for Fe (III) adsorption on PYCS.

Sample	Δ*S* (J/mol·K)	Δ*H* (kJ/mol)	Δ*G* (kJ/mol)			
			303 K	313 K	323 K	333 K
PYCS	36.49	8.14	−2.91	−3.27	−3.64	−4.00

**Table 5:** Metal concentration (mg/L) in strongly acidic wastewater from coking plant.

Fe (III)	As (III)	Pb (II)	SO_4_^2−^	pH
484 ± 10	0.7 ± 0.1	0.6 ± 0.1	1260	0.5

## Data Availability

Compound data sets are publicly available. Samples are available from the authors.

## Sample Availability

Not available.
